# The Effects of Microgravity on the Structure and Function of Cardiomyocytes

**DOI:** 10.3390/biom15091261

**Published:** 2025-08-30

**Authors:** Luis Fernando González-Torres, Daniela Grimm, Marcus Krüger

**Affiliations:** 1 Department of Microgravity and Translational Regenerative Medicine, Otto-von-Guericke University, 39106 Magdeburg, Germany; luis.gonzaleztorres@med.ovgu.de (L.F.G.-T.); daniela.grimm@med.ovgu.de (D.G.); 2 Research Group “Magdeburger Arbeitsgemeinschaft für Forschung unter Raumfahrt- und Schwerelosigkeitsbedingungen” (MARS), Otto-von-Guericke University, 39106 Magdeburg, Germany; 3 Department of Biomedicine, Aarhus University, 8000 Aarhus, Denmark

**Keywords:** cardiomyocytes, microgravity, spaceflight, tissue maturation

## Abstract

Spaceflight and microgravity (μg) environments induce numerous cardiovascular changes that affect cardiac structure and function, and understanding these effects is essential for astronaut health and tissue engineering in space. This review compiles and analyzes over 30 years of research on the impact of real and simulated μg on cardiomyocytes. A comprehensive literature search was conducted across five databases, and 62 eligible studies involving cardiac cells under μg or spaceflight conditions were compiled and analyzed. Despite the great heterogeneity in terms of cardiac model, microgravity platform, and exposure duration, multiple studies consistently reported alterations in Ca^2+^ handling, metabolism, contractility, and gene expression. Three-dimensional human-induced pluripotent stem cell-derived cardiomyocyte (HiPSC-CM) models generally showed enhanced tissue maturation and proliferation parameters, suggesting potential therapeutic benefits, while 2D models mostly exhibited stress-related dysfunction. In vivo simulated microgravity studies, such as the hindlimb unloading (HU) model, show structural and functional cardiac remodeling, and real μg studies confirmed various effects seen under the HU model in multiple rodent species. Thus, μg exposure consistently induces cardiac changes at the cellular and molecular level, while model choice, microgravity platform, and exposure duration critically influence the outcomes.

## 1. Introduction

**Figure 1:**
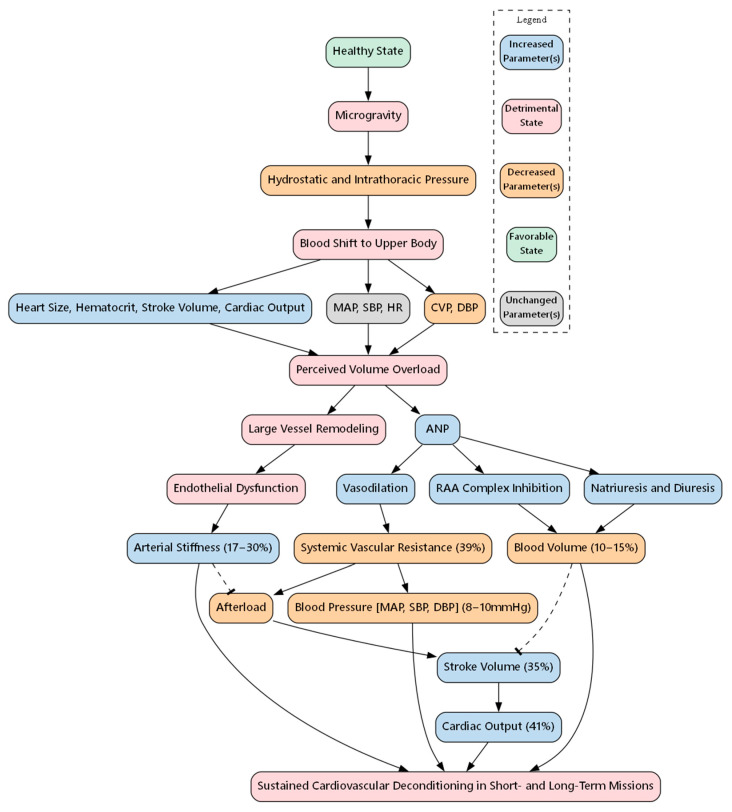
Systemic cardiovascular changes experienced during spaceflight. CVP, Central venous pressure; DBP, diastolic blood pressure; MAP, mean arterial pressure; SBP, systolic blood pressure; HR, heart rate; ANP, atrial natriuretic peptide; RAA, renin–angiotensin–aldosterone. Arrows indicate direction of causal relationships, dotted lines indicate an inhibitory effect.

A range of systemic cardiovascular changes are experienced by astronauts during long-term space missions. Mainly, the absence of gravity and the cosmic radiation that astronauts are exposed to in orbit can induce the heart, and the cardiovascular system in general, to adapt to the new environment and undergo long-term structural and functional changes that result in pathological conditions. There are other contributing physiological and psychological stressors, which include isolation and circadian rhythm alterations, as well as sleep, dietary, and physical activity constraints. However, microgravity (μg) plays a key role in remodeling the cardiovascular system of astronauts in space, as demonstrated in Figure 1 [1].

Initially, as the absence of gravity causes the hydrostatic pressure gradient and the intrathoracic pressure to decrease, the blood shifts from the lower to the upper part of the astronaut’s body [2]. This causes an initial increase in heart size, hematocrit levels, stroke volume (SV), and cardiac output (CO) and a decrease in central venous pressure (CVP) and diastolic blood pressure (DBP), while mean arterial pressure (MAP), systolic blood pressure (SBP) and heart rate (HR) remain unchanged [3,4,5]. These changes trigger a response from central cardiovascular regulatory mechanisms to reduce the perceived volume overload. Atrial natriuretic peptide (ANP) secretion increases as a response to the perceived volume overload, inducing vasodilation and inhibiting the renin–angiotensin–aldosterone system [1,4]. This results in a cardiovascular deconditioning effect in which there is an approximate 10–15% reduction in blood volume and a ~39% reduction in systemic vascular resistance (SVR) that is sustained during short- and long-term spaceflight missions, along with increases in SV and CO [1,4,6]. Sustained changes also include an increase in arterial stiffness due to large vessel remodeling and endothelial dysfunction, along with decreases in MAP, SBP, and DBP [4].

Cardiac structural changes have also been reported. Some known structural changes include, for example, the left atrial enlargement reported after six months in space [7]. This change was reversed weeks later after returning to Earth, but it may increase the risk of atrial fibrillation, which tends to present itself at a younger age among astronauts [7]. Additionally, nonfatal arrhythmias have been documented, including ventricular tachycardia and atrial and ventricular premature contractions, although their causes remain unknown [8,9,10]. Otsuka et al. showed altered HR variability (HRV) patterns in astronauts during long-term spaceflight. However, HR remained unchanged [11]. As the heart requires less force to send blood upwards and maintain arterial pressure, cardiac atrophy has been hypothesized to rise, especially since skeletal muscle atrophy has been widely reported [1,4]. Nonetheless, long-term (>6 months) spaceflight data on this subject have not been recorded, and reported decreases in left ventricular mass during short-term missions have been attributed mostly to dehydration and plasma volume loss, not cardiac atrophy [12,13,14]. Finally, the most common problem among astronauts arising from cardiovascular deconditioning is postflight orthostatic intolerance (POI) [4,15]. POI incidence has been found to be ~20% and ~80% after short- and long-term missions, respectively. It is characterized by the inability of the body to compensate for arterial blood pressure changes during postural changes (i.e., standing up), and it includes symptoms such as dizziness, fatigue, headache, hypotension, nausea, sweating, and vomiting [15].

Although medicinal, nutritional and exercise countermeasures have been developed, several factors affecting cardiovascular health in space persist. Further research is needed to elucidate appropriate interventions during specific time points that boost human adaptability in space while minimizing the harmful effects of spaceflight [16,17]. Thus, there are numerous cardiovascular changes that need to be addressed and further understood to ensure for the health of astronauts during both short- and long-term missions. For this reason, cardiac cell research under μg has been an ongoing effort over the past 30 years [18]. Understanding the cellular and molecular mechanisms underlying these cardiovascular changes may assess the development of strategies to treat or even prevent these spaceflight-induced effects. It may also help to develop more robust cardiac models under μg, which may elucidate previously unknown changes in cardiac structure and function and may discriminate between the cardiovascular effects caused by μg and those caused by other spaceflight variables, such as radiation, isolation, etc.

Tissue engineering research may also benefit from cardiac cell research under μg. Some data suggest that exposure to μg conditions may contribute to the maturation of cardiac tissue constructs developed from human-induced pluripotent stem cell-derived cardiomyocytes (HiPSC-CMs) [19,20,21]. These enhanced tissue constructs may result in the development of cardiac grafts or patches and may be used to treat heart conditions both in space and on Earth. The aim of this review is to summarize the findings of over 30 years of cardiac cell research under μg or spaceflight conditions. Given the heterogeneity of experimental setups and reported outcomes, the review categorizes the results in terms of type of μg model, cardiac model, exposure duration, and structural and functional effects observed. Using these categories helps illustrate the advantages, limitations, and future directions of each μg platform and model. Furthermore, it helps assess the translatability from simulated μg (s-μg) to real μg.

## 2. Materials and Methods

**Figure 2:**
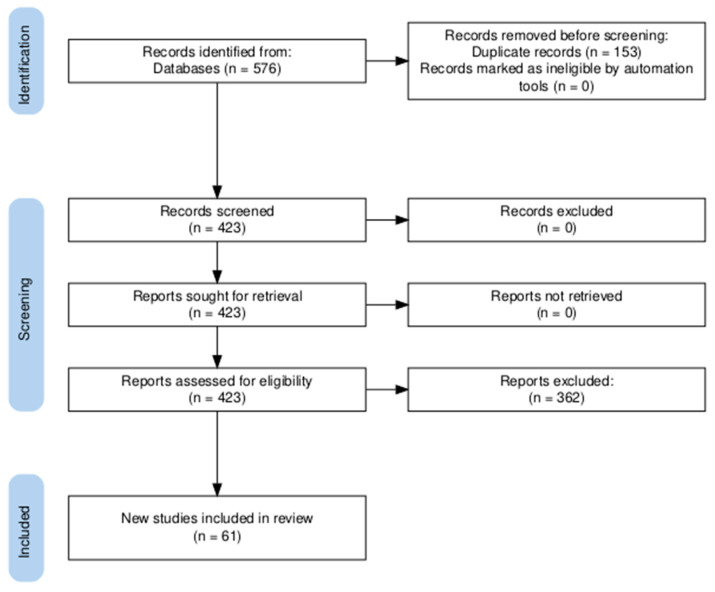
PRISMA flow diagram detailing the literature search and screening process.

This review was conducted in accordance with the PRISMA guidelines (Figure 2). A protocol was prepared prior to search; however, it was not registered in a public database. The comprehensive literature search was performed across PubMed, Scopus, Embase (ScienceDirect), Google Scholar, and BIOSIS Previews. The search strategy consisted mainly of the keywords, “Cardiomyocytes AND (microgravity OR spaceflight)” unless stated otherwise in Table 1. The last search was conducted on 21 April 2025. A total of 576 records were retrieved across all databases. All retrieved records were exported to Zotero, and duplicates were removed. A total of 423 articles remained after duplicate removal. Titles and abstracts were screened based on the eligibility criteria (Table 2). After applying the exclusion criteria, 61 articles were included in the final analysis.

## 3. Effects of Spaceflight and Real Microgravity on Cardiac Models

Research under real μg conditions is achieved through parabolic flights on Earth, suborbital rocket flights, or through access to satellites, the International Space Station (ISS), or the Chinese Space Station (CSS) [22]. Given the high costs and long planning associated with them, there are only a few opportunities to perform experiments under real μg conditions. A total of 19 articles with cardiac models under real μg were identified for this review (Supplementary Table S1). Studies performed under these conditions have elucidated short- and long-term changes in the physiology of 2D and 3D cardiac models with potential applications in the field of disease modeling, stem cell development, tissue engineering, and regenerative medicine [19,20]. Additionally, animal studies performed on the ISS have shown structural and functional changes on cardiac tissue at the cellular and molecular level that are not possible to observe or analyze in humans.

### 3.1. In Vitro Studies

#### 3.1.1. Primary Cardiomyocytes

Only one study involving primary cardiomyocytes under real μg was found during the literature search. Isolated cardiomyocytes from neonatal Wistar rats were 2D cultured for ~5 days aboard the Shenzhou-6 spacecraft [23]. The cells showed microtubule disassembly, a reduction in ANP secretion, and disordered contraction with a reduced number of spontaneous beating sites. While this is an appropriate first approximation to understand what happens to isolated primary cardiomyocytes under real μg, future work may benefit from more physiologically relevant models with human samples that better resemble the systemic changes that astronauts undergo in space. Introducing new technologies, such as microfluidic channels with appropriate flow and pressure rates that mimic those experienced by astronauts at different timepoints, may help in the discovery of new structural and functional changes that align more with what is actually reported at the systemic level. Co-culturing with other relevant cell lines (e.g., cardiac fibroblasts and endothelial cells) and adding small molecules (e.g., ANP) that are known to communicate with cardiomyocytes during spaceflight may also enhance the significance of the findings and result in a relevant disease model on which different therapeutic strategies can be tested.

#### 3.1.2. Human-Induced Pluripotent Stem Cell-Derived Cardiomyocytes

Two-dimensional HiPSC-CMs cultured for 6 days aboard the CSS showed an impairment in thiamine utilization, affecting the tricarboxylic acid (TCA) cycle and reducing ATP production [24]. In addition to this, metabolomic and KEGG pathway analyses showed 13 dysregulated metabolites mainly involved in the sulfur relay system, thiamine metabolism, ABC transporters, and vitamin digestion and absorption. Additionally, there was a decrease in sarcomere length, cardiomyocyte size, cTnT content, contractile function and slower Ca^2+^ cycling. mRNA expression also showed changes in cardiomyocyte-specific genes such as *TNNI1*, *TNNI3*, *ATP2A2*, and *CACNA1C*. Finally, thiamine supplementation under s-μg was shown to improve ATP production, Ca^2+^ handling, and restore TCA cycle function and sarcomere structure. These data suggest that 2D HiPSC-CMs experience significant structural and metabolic changes under real μg after 6 days, and thiamine may play an essential role [24]. A similar study with 2D HiPSC-CMs cultured on the ISS for 5.5 weeks also found alterations in Ca^2+^ handling parameters, including increases in the transient decay tau and the standard deviation of beating intervals, which suggest decreased Ca^2+^ recycling rate and beat irregularity, respectively [25]. However, no effect on sarcomere structure, length, or regularity was found. A transcriptome analysis revealed changes in genes related to hypertrophy (*MEF2D*, *HDAC10*, *HDAC4*, *HDAC8*) and Ca^2+^ handling and contraction (*TNNT2*, *TNNI1*) [25]. Finally, 2D HiPSC-CMs were cultured aboard a parabolic flight during the 66th Parabolic Flight Campaign of the European Space Agency. The results showed a significantly elevated beating rate after the 15th parabola that persisted until the last parabola, and even one hour after the flight, compared to 1 g controls [26].

On the other hand, HiPSC-CMs cultured for 3 days and 3 weeks as 3D cardiac spheroids aboard the ISS showed an increase in cell size, spheroid size, Ca^2+^ handling kinetics, as well as proliferation and differentiation markers [19,20]. In association with the previously mentioned study, genes related to the TCA cycle were upregulated after 3 days [20]. Proliferation markers such as *CCND1*, *CCND2*, *IGF2*, and *TBX3* increased under real μg, along with the percentage of Ki67-positive cells, for both time points. In addition, cardiac function-related gene expression, including *CCBE1*, *IGFBP5*, *MYL2*, *MYH6*, *MYH7*, *TNNI3*, and *CASQ2*, was upregulated under real μg for both time points. Meanwhile, *MYL7*, *TNNT2*, *ATP2B4*, and *TNNI1* were elevated only after 3 weeks. Additionally, 3-week samples showed a decrease in extracellular matrix (ECM)-related genes, such as *ITIH5*, *COL4A4*, *PTPR21*, *COL26A1*, and *HPX* [19]. Real μg samples also showed an increase in peak amplitude, maximum rise slope, and maximum decay slope and a decrease in time to peak and half-width, indicating more mature Ca^2+^ handling kinetics [20]. Another study cultured HiPSC-CMs as 3D cardiac spheroids aboard the ISS for 8 days [27]. While contractility and Ca^2+^ transients remain unchanged, proteins related to cell survival, proliferation, and metabolic pathways were upregulated (UGT2A3, EEF2K, KRT13, PRIM1). Moreover, genes related to metabolism, mitochondria, and cardiac development and structure were upregulated (*TNNT2*, *PLCB2*, *CPNE5*, *CDH17*, *SFRP5*, *PLA2G4F*, *GPAT2*) [27].

A different study cultured HiPSC-CMs inside a decellularized porcine myocardial extracellular matrix (dECM) scaffold with reduced graphene oxide (rGO) and analyzed them aboard the ISS for 30 days [28]. Contrary to previous results, 3D cultures within this scaffold aboard the ISS showed a reduced contractile twitch force, downregulation of contractility-related genes (*MYL7*, *MYL3*, *MYH7B*, *TNNI3*, *RYR2*, *ATP2A2*, *TTN*), and an increased arrhythmic activity. Structural damage, including shortened sarcomeres and fragmented mitochondria, and upregulation of inflammation and oxidative stress-related genes (*NOXO1*, *GGTLC3*, *IL18*, *TGFB2*) were also observed [28]. This shows that not all 3D culture techniques under μg enhance tissue maturation markers and properties.

Thus, clear differences in results between 2D and 3D cardiac model under real μg have been observed. While studies using 2D cardiac models reported negative effects such as impaired metabolism, gene dysregulation, and reduced contractile function, studies using 3D cardiac models reported improvements in Ca^2+^ handling parameters, increased proliferation, and upregulation of cardiac tissue maturation markers. This sharp contrast in results illustrates the importance of choosing an adequate model given the role that μg may play either as a stressor or as a tissue maturation promoter depending on the model. Moreover, as in the case with primary cardiomyocytes, this initial work with 2D models reveals important changes that isolated HiPSC-CMs may experience under spaceflight or real μg conditions. However, if it is desired to understand the cardiovascular changes that astronauts’ experience during spaceflight at the cellular and molecular level, improved physiologically relevant models are required.

On top of this, the exposure of 3D cardiac models to real μg conditions resulted in tissue maturation characteristics that may be beneficial for the fields of tissue engineering, regenerative medicine, and stem cell therapy. HiPSC-CMs still face many obstacles that hinder their application in heart myocardial injury therapy, including limited proliferative capacity and a fetal-like immature phenotype [29,30]. Multiple strategies have been developed to enhance the maturation and proliferative capacity of HiPSC-CMs. Some of these strategies include the use of biophysical cues such as electrical and mechanical stimuli, biomaterials, and scaffolds that mimic ECM behavior as well as 3D systems co-cultured with cardiac fibroblasts, endothelial cells, or biomolecules that induce proliferation- or maturation-related pathways [29,31]. The use of μg environments in combination with other strategies could be a useful tool to achieve cardiac tissue maturation and increased proliferative capacity on HiPSC-CMs. However, as exemplified with the HiPSC-CMs cultured on the dECM-rGO scaffold, not all combinations may result in enhanced cardiac tissues and some may even be detrimental to them [28]. Additionally, if it is desired to exploit their potential benefits in patients back on Earth, it is necessary to elucidate how long these proliferation and tissue maturation parameters remain enhanced post μg exposure and whether they are useful for the field of regenerative medicine and cell therapy. Nonetheless, further testing and validation in collaboration with regulatory agencies may result in relevant therapeutic solutions.

#### 3.1.3. Cardiovascular Progenitor Cells and Embryonic Stem Cells

Human Islet-1+ cardiovascular progenitor cells (CPCs) from adult and neonatal sources were 2D cultured aboard the ISS for 12 and 30 days [32,33]. After 12 days, neonatal CPCs showed an increased expression of markers related to an earlier developmental state, such as *WNT5A* and *ISL1*. Ca^2+^ signaling-related genes were also upregulated, such as *RYR2*, *CACNA1S*, and *CAMK2A* on day 12 and *PLCG1*, *PRKCA*, *CAMK2A* on day 30. Gene and protein expression changes confirmed the activation of protein kinase C α (PKCα) and Akt in neonatal CPCs after 30 days, furthering the impact of real μg on Ca^2+^-related signaling [33]. RNA sequencing revealed an increase in cardiac commitment markers *MESP1* and *NKX2-5* regardless of age after 30 days, as well as an elevation in stemness-related markers. Furthermore, pathway analysis revealed that the Wnt/β-catenin, Ca^2+^ signaling, Notch, ERBB, and Hippo pathways were induced after 30 days regardless of age. Finally, independent of age, genes related to specific biological processes such as cell cycle progression, proliferation, differentiation, heart development, oxidative stress protection, and focal adhesion were induced after 30 days [32]. Similar results were obtained with mouse embryonic stem cells (mESCs) flown aboard the Space Shuttle Discovery and differentiated into embryoid bodies (EBs) for 15 days [34]. Under real μg, the EBs expressed a higher level of stemness-related markers and showed a reduced capacity to differentiate into other tissue types and to express terminal differentiation markers. They also showed alterations in cell cycle as well as p53, Notch, and Wnt signaling pathways and exhibited twice the potential to differentiate into beating cardiomyocyte clusters upon returning to Earth [34]. Moreover, EBs derived from mouse iPSCs flown for 14 days also showed a faster and more robust cardiomyocyte differentiation than 1 g controls [35].

Thus, studies using CPCs and mESCs under real μg reported changes related to Ca^2+^ signaling along with increases in proliferation and stemness-related markers associated with an earlier developmental stage [32,33,34]. These results have been in turn associated with an increased therapeutic potential after administration of similar cell types in heart failure patients [36,37,38,39]. However, as in the case of 3D HiPSC-CMs, this potential application requires further testing and validation in collaboration with regulatory agencies. Future treatment of preclinical heart failure models with this type of μg-exposed cells may produce promising results. Meanwhile, the studies with mouse stem cells showed promising results related to a faster and more robust differentiation into cardiomyocytes. Future work could also concentrate on the potential to exploit real μg as a tool in differentiation protocols to help advance the field of tissue engineering and stem cell research.

### 3.2. Animal Studies

While considering their physiological and ethical limitations, animal studies under spaceflight conditions still offer the possibility to study how systemic cardiovascular changes affect the organism at the tissue, cellular, and molecular level. Multiple species have been studied in space to understand the impact of μg and/or cosmic radiation on cardiac tissue. For example, *Drosophila melanogaster* samples were aboard the ISS using vented fly boxes for 30 days [40]. The results showed cardiac constriction, reduced heart chamber size, diminished cardiac output, and myofibrillar and ECM remodeling in the flight samples. A gene expression analysis demonstrated the reduced expression of actin, myosin, and ECM components [40]. Additionally, Wistar male rats were hosted aboard the COSMOS 2044 Biosputnik satellite for 14 days. They showed a reduction in the cross sectional area of myofibrils for the papillary muscles compared to controls, but not for the ventricular muscles [18]. Finally, Sprague Dawley pregnant rats and their fetuses were also analyzed after 11 days of spaceflight. These rats exhibited a tendency toward increased ANP biosynthesis compared to ground controls [41]. Nonetheless, these are the only studies involving these species and the heterogeneity of the data makes it difficult to reach any significant conclusions.

#### 3.2.1. Male C57BL/6 Mice

Male C57BL/6 mice are the most widely studied animals in space. Mice flown aboard the Russian spacecraft “BION-M1” for 30 days experienced skeletal muscle atrophy and a reduction of sarcomere-related proteins (titin, nebulin) in skeletal muscles [42]. However, no cardiac atrophy was reported and there was an increase in titin mRNA and phosphorylation levels in addition to disruption in cardiac sarcomeric structure. A study from the same mission also reported decreases in cytoplasmic fraction β-actin and membranous fraction α-actinin 4 in cardiomyocytes from the left ventricle. mRNA levels of β-actin (*ACTB*) and α-actinin 4 (*ACTN4*) also decreased, while α-actinin 1 (*ACTN1*) showed an increase [43]. It must be noted that the samples were euthanized within 13 and 16.5 h after the BION-M1 landing, and the effects due to μg may have been overlapped by the hypergravity experienced during the descent and by the early readaptation process to the Earth’s gravity [42,43]. The NASA’s Rodent Research missions performed on the ISS have addressed this issue, and the samples were euthanized on-orbit, along with the tissue dissection and preservation [44]. However, results from these missions have not addressed cardiac tissue effects.

Male C57BL/6 mice have also flown on parabolic flights to study the role of adenylyl cyclase type 5 (AC5) under real μg [45,46]. These results indicate that mice treated with NKH477 (an AC5 selective activator) show a more stable HR and HRV during the flight in comparison to the control group and a group of mice treated with vidarabine (an AC5 inhibitor). Moreover, treatment with NKH477 increased the ratio of high frequency power, a marker of parasympathetic tone, suggesting that pharmacological activation of AC5 may help to maintain the parasympathetic tone and stabilize the heart rate under altered gravity [45]. Similarly, transgenic C57BL/6 mice with overexpression of AC5 exhibited a more stable HRV than wild type and AC5 knockout mice, further reinforcing the idea that AC5 may play a role in stabilizing HR during altered gravity conditions.

#### 3.2.2. Current Insights, Limitations, and Future Directions for Animal Studies in Space

Perhaps, the main insight from the animal studies performed under real μg is that cardiac tissue indeed experiences structural remodeling during spaceflight. However, the circumstances and extent under which cardiac remodeling takes place are not completely clear. This remodeling is reported in terms of myofibrillar and ECM remodeling in the fruit fly model, reduction in myofibril cross-sectional area in Wistar rats, and sarcomeric structure disruption and cytoskeletal alterations in C57BL/6 mice [18,40,42,43]. However, once again, the extensive range of animal species studied under μg or spaceflight conditions, in addition to differences in experiment duration and measured outcomes, make it difficult to draw more definitive conclusions. Moreover, species-specific differences in cardiac physiology limit the extrapolation of findings from animal models to human physiology, complicating their use in biomedical research [47]. Thus, future animal studies should be more aware of experiment duration, measured outcomes, and species-specific differences in order to produce translatable and applicable results to human physiology.

## 4. Effects of Simulated Microgravity Conditions on Cardiac Models

Given the high costs and extensive planning associated with spaceflight and real μg platforms, ground-based platforms have been developed over the years to simulate spaceflight conditions, such as μg and radiation, at a fraction of the cost. Even with their respective limitations, platforms such as the rotating wall vessel (RWV), the clinostat, and the random positioning machine (RPM) offer different ways to produce what is known as s-μg [48]. A total of 16 studies regarding the use of such platforms with cardiomyocytes were identified (Supplementary Table S2). Either by making the gravity vector average zero or by keeping the models in constant suspension (weightlessness), these platforms can produce preliminary results that may guide future experiments with cell or tissue models under real μg. By doing this, space-related institutions and researchers can make informed decisions with regards to which models are worth the costs and logistics related to spaceflight.

Furthermore, the hindlimb unloading (HU) model is the most widely utilized in vivo ground model to induce the effects of μg on musculoskeletal and cardiovascular systems [49]. The HU model is characterized by suspending a rodent at a head-down tilt angle of ~30° so that the hindlimbs are unloaded and unable to bear any weight, while the forelimbs remain on the floor and permit movement and access to food and water. The aim of this model is to cause a cephalad fluid shift, to mimic the mechanical unloading experienced by astronauts in space, and to study the pathophysiological changes experienced at the cellular and molecular level [49,50]. This model also allows for longer term studies involving in some cases up to 8 weeks of s-μg exposure [51].

### 4.1. In Vitro Studies

#### 4.1.1. Primary Cardiomyocytes

Primary cell lines have been exposed to s-μg mostly to elucidate the negative effects it may have on them and to potentially develop countermeasures against these effects. For example, 2D cultured cardiomyocytes derived from 2-day-old Wistar rats were exposed to s-μg using a 2D clinostat for up to 2 days [52]. Under s-μg, nitric oxide levels were found to increase, as well as inducible nitric oxide synthase mRNA and protein expression. Staurosporine (a nonselective protein kinase inhibitor) and calphostin C (a selective PKC inhibitor) were able to attenuate these changes and demonstrate that PKC plays a role in NO regulation [52]. A different study used cardiomyocytes derived from the ventricles of 2-day-old Sprague Dawley rats and attached them to the surface of microcarrier beads for up to 5 days inside a RWV [53]. The data showed that s-μg caused a reduced protein synthesis, activation of unfolded protein response, and upregulation in mitochondrial maintenance-related proteins such as mortalin and AFG3L2. These findings suggested that the cells prioritize mitochondrial integrity at the expense of protein synthesis under s-μg [53]. A similar study let the cardiomyocytes attach to the microcarrier beads and analyze them after 6 days in a RWV [54]. The cells exhibited a 3D multilayered growth pattern not seen in the 1 g condition. Additionally, elevated levels of NAD-dependent cytochrome-c reductase and slightly reduced levels of creatine kinase suggested an increase in oxidative metabolism [54].

Thus, studies with primary cardiomyocytes under s-μg have revealed significant changes related to NO regulation, mitochondria, reduced protein synthesis, and increased oxidative metabolism. Given that there is only one study regarding primary cardiomyocytes under real μg, it is difficult to assess how well the results in this section translate to the real environment. Nonetheless, both results showed signs of stress that may interfere with the normal function of cardiomyocytes. As stated before, work with primary cardiomyocytes may benefit from more complex and robust models that mimic the mechanical and chemical stimuli that the heart of astronauts are exposed to while in orbit. It should also be noted that the cardiac cells of astronauts are not directly exposed to μg, but are instead exposed to altered shear and compression forces arising from the effects that μg has on the cardiovascular system as a whole [4]. Complementing the use of cardiac cells with technologies that mimic organ or tissue function may provide more valuable results than studies with cells and μg platforms alone. Microfluidic systems such as the organ-on-a-chip can provide cardiac models with controlled perfusion, electrical stimulation, shear forces, and other mechanical cues that better mimic physiological conditions [55,56].

#### 4.1.2. Stem Cell-Derived Cardiomyocytes

HiPSC-CMs have been widely studied under s-μg conditions, and the results offer similar conclusions as the real μg experiments previously discussed. Two-dimensional HiPSC-CMs cultured for 2 days on a RWV showed an increase in reactive oxygen species (ROS), as well as impaired mitochondrial function, slower Ca^2+^ influx, and reduced contraction velocity [57]. In addition to this, the chromatin organization and 3D genome architecture were altered following s-μg. On the other hand, 3D HiPSC-CMs cultured for 7 and 14 days on the RPM revealed enhanced tissue maturation features [58]. After 14 days on the RPM, the cardiac spheres grew in terms of diameter in comparison to their 1 g counterparts, showing signs of proliferation. Additionally, after 7 days, there was an improvement in Ca^2+^ transient properties, as shown by an increase in the amplitude (F/F0) of Ca^2+^ transients, maximum rise slope and maximum decay slope. The mitochondrial characteristics were also improved after 7 days, as evidenced by the increased mitochondrial content and enhanced mitochondrial potential, respiration, and ATP production. Furthermore, sarcomere and z-disc length were elevated after 7 days [58]. Finally, 3D HiPSC-CMs were cultured on a scaffold of poly lactic-co-glycolic acid-aligned nanofibers inside a RWV for 1, 3, and 5 days [59]. The study found time-dependent structural changes and an increased expression of cardiac-specific protein markers (β-MHC and connexin-43) after day 3, suggesting tissue maturation.

HiPSC-derived cardiac progenitor spheres were cultured on the RPM on day 5 of their differentiation process for 3 days, after which they were placed in 1 g conditions to finish the process (day 21) [21]. Compared to 1 g controls, cardiomyocyte yield and viability increased, along with an upregulation of cardiomyocyte structural genes (*MYH6*, *MYH7*, *MYL2*, *MYL7*, *TNNI1*, *TNNI3*) and Ca^2+^ handling genes (*ATP2A2*, *CASQ2*, *RYR2*, *SLC8A1*). Regarding Ca^2+^ handling, the maximal upstroke and decay velocities were also faster, and the time from the peak to 50% decay was shorter, suggesting enhanced Ca^2+^ reuptake [21]. Another study used mouse models (including cKit^CreERT2/+^, Isl1nLacZ, Wnt1-Cre reporter alleles) to label and trace specific cardiac cells and to induce PSCs from them [60]. The study consisted of placing differentiation day 4 EBs in a RWV and continuing their differentiation process for 6 days under s-μg, after which they would be brought back to 1 g conditions to complete differentiation (day 21). Results showed that s-μg exposure favors the growth of mesodermal cardiac progenitors over neural crest derived autonomic neurons and cardiomyocytes; however, there was a reduction in the rate of spontaneously beating Ebs on day 10 of the differentiation process [60]. Finally, mESCs were also exposed to s-μg using a 2D fast rotating clinostat during their differentiation process [61]. This time, mESCs were exposed to s-μg for 3 days since day 1 and continued the differentiation process for 7 more days. In this case, cardiac-specific genes such as *Tnnt2*, *Rbp4*, *Tnni1*, *Csrp3*, *Nppb*, and *Mybpc3* were downregulated after 10 days, in correlation with the observed decrease in EB beating rate [61].

As observed from the results, HiPSC-CMs and other stem cells have been used widely in μg research. Consistent changes in Ca^2+^ handling, contraction, and cardiac markers have been reported under both real and s-μg environments independently from the cardiac model used [19,20,24,25]. The signs of cellular stress and the increase in proliferation and tissue maturation markers observed in 2D and 3D HiPSC-CMs, respectively, align with those found under real μg conditions. While taking into consideration the physical and biological limitations that keep s-μg platforms from fully mimicking the real μg environment, these results exemplify their ability to partially replicate real μg environments and to serve as a prescreening tool that allows for the optimization of model conditions before committing to costly and lengthy spaceflight experiments. In the case of studies where the goal is to enhance the cardiomyocyte differentiation process, close attention should be paid to the cell line, the differentiation protocol, and the duration of μg exposure since changes in these factors have produced very different outcomes, as shown on the results section.

#### 4.1.3. Immortalized Cardiomyocyte Cells

Other cells lines exposed to s-μg conditions include H9C2 and HL-1 cardiomyocytes. HL-1 cardiomyocytes are derived from female C57BL/6 mouse atrial tumors and are relevant for biomedical science research given their ability to serially divide while maintaining spontaneous contraction and a differentiated cardiac phenotype [62,63]. After being exposed to s-μg using a 2D clinostat for 2 days, 2D-cultured HL-1 cardiomyocytes exhibited increased Ca^2+^ handling parameters such as basal cytosolic Ca^2+^, Ca^2+^ oscillations, and Ca^2+^ transients, along with a decrease in cell size and MHC-α protein and mRNA expression levels [64]. It was also found that the phosphorylation of Ca^2+^/calmodulin-dependent protein kinase II (CaMKII) and histone deacetylase 4 (HDAC4) was increased, as well as ANP and brain natriuretic peptide (BNP) mRNA expression levels, suggesting that altered Ca^2+^ signaling has downstream effects on cardiac remodeling pathways. To further support this claim, the use of small interfering RNA (siRNA)-CaMKII prevented the phosphorylation of HDAC4 and cell size decrease, indicating possible CaMKII/HDAC4 pathway involvement in cardiac remodeling [64]. Another study with HL-1 cells cultured under the same s-μg conditions for 3 days reported alterations in the cytoskeleton, adhesive system, and proliferative capacity, in addition to an increase in apoptosis [65].

H9C2 cells are non-beating cells derived from embryonic rat ventricular tissue that proliferate and retain similar morphological and electrophysiological properties as primary cardiomyocytes [63]. When exposed to s-μg on an RPM for up to 4 days, 2D-cultured H9C2 cells showed morphological changes characterized by increases in cell height and actin filament mean length, together with altered mitochondrial branching complexity [66]. S-μg also affected the cells in terms of increased levels of cytosolic Ca^2+^, medium glucose, medium lactate, ROS, and mitochondrial ^•^O_2_^−^, along with decreased mitochondrial potential and metabolic activity. Intervention with 1mM of N-acetyl-cysteine, an antioxidant, prevented the increase in cytosolic Ca^2+^, medium glucose, medium lactate, ROS, and mitochondrial ^•^O_2_^−^ after 24 h [66]. Another study found that H9C2 cells attached on the surfaces of microcarrier beads and cultured on a RWV for 3 h induce the nuclear translocation of NF-κB subunit p65; however, further research is needed to understand its role under s-μg [67].

Thus, the short-term exposure of these immortalized cell lines to s-μg has revealed increases in Ca^2+^ signaling parameters not previously seen in the other 2D cardiac models under real and s-μg, along with other changes related to cellular stress and metabolism. Nonetheless, the studies with HL-1 cells revealed the possible mechanisms through which s-μg exposure results in cardiac remodeling. The involvement of the CaMKII/HDAC4 pathway in this regard has been further supported and expanded by HU experiments, which are presented below (see Section 4.2.4). However, the immortalized cells used in this study were exposed to s-μg for only 2 days, while the reported HU experiments lasted for at least 2 weeks. This reflects the need for future work to set appropriate experiment durations that align with the typical duration of spaceflight missions or with the times at which systemic parameter are measured in astronauts while in orbit.

#### 4.1.4. Simulated Space Radiation

Analyzing the cardiac cell response to simulated space radiation (SSR) is also important to understand the possible behavior of cardiac cells during spaceflight. Cardiomyocytes isolated from male C57BL/6NT mice were irradiated with either 900 mGy of protons or 150 mGy of ^56^Fe ions [68]. The study found that radiation, specially that coming from protons, activated the *FYN* gene pathway, which is involved in ROS reduction, along with a downregulation in the ROS pathways. Additionally, gene expression changes were found even 28 days after exposure, indicating the long-term effects of SSR on cardiomyocytes [68].

Another study exposed primary cardiomyocytes derived from chicken embryos to SSR using heavy ions (iron, titanium, and carbon) and analyzed their response up to 24 h after exposure [69]. Results indicated efficient DNA damage repair after irradiation, even at high doses, including almost a complete recovery of DNA double-strand breaks within 24 h. Other changes included dose- and ion-dependent reductions in proliferation and beating rate [69]. Finally, another SSR study encapsulated HiPSC-CMs into a fibrin hydrogel and exposed them to neutron (1 Gy) or photon (4 Gy) radiation [70]. Three weeks post-exposure gene expression analysis showed an increase in stress- and hypertrophy-related genes. Moreover, there was an increase in force generation and contractility [70]. Thus, mixed results have been found depending on the cell line and the type and intensity of SSR utilized. Nonetheless, the changes herein reported indicate that SSR should be considered in future studies given its potential impact on the structure and function of cardiomyocytes. The combination of SSR with s-μg may help develop relevant models whose results translate better to those obtained during experiments on the ISS or other spaceflight platforms.

### 4.2. Hindlimb Unloading

Multiple rodent species have been used along with the HU model, producing a variety of results that need to be categorized and properly understood. It was the most found model in our literature search, with 26 out of 61 articles reporting its use (Supplementary Table S3).

#### 4.2.1. Wistar Rats

First, male Wistar rats exposed to HU experienced an increase in ANP plasma levels only 1 h after suspension. However, ANP reversed to baseline after another hour, and remained constant for the next 4 h [71]. On the other hand, ventricular cardiomyocytes isolated from male Wistar rats after 7 days of HU showed a dysregulated gene expression pattern in mechanically gated (*TRPM7*, *TRPP1*, *TRPP2*, *PIEZO1*, *TMEM63A*, *TMEM63B*, *TRPV2*) and mechanosensitive (*KCNK2*, *KCNK3*, *KCNJ11*, *KCNJ8*, *SCN5A*, *CACNA1C*, *KCNQ1*) ion channels [72]. They also revealed gene expression changes in phosphodiesterase (*PDE2A*, *PDE3A*, *PDE4A*, *PDE4B*), soluble guanylate cyclase (*GUCY1A1*, *GUCY1A2*, *GUCY1B1*), and adenylate cyclase (*ADCY5*, *ADCY6*) isoforms, which are involved in pathways that regulate mechanosensitive and mechanically gated channels [73]. Another study with the same model reported an increase in transversal stiffness of the contractile apparatus in multiple locations after 3 days of HU [74]. This rise remained elevated until day 14 of HU but reversed to control levels after a 7-day reloading period. Respiration rate parameters, desmin protein content, and membranous γ-actin protein content were also elevated after just 1 day of HU. These levels remained elevated throughout 14 days of HU and reversed to control levels after 7 days of reloading. Meanwhile, cytoplasmic α-actinin-4 protein content was elevated from day 1 until day 14 of HU and did not reverse to control levels after the 7-day reloading period, which suggests that some effects are irreversible [74].

A long-term study exposed male Wistar rats to 24 days of HU and found a 5% increase in the left ventricle cardiomyocyte cross sectional area, along with an 11.5% increase in the relative number of capillaries per cardiomyocyte, indicating a possible compensatory hypertrophy effect [75]. In addition, there was an increase in the intramitochondrial junctions (IMJ) after the 24 days [76]. A different study with the same species found a decrease in left ventricle cardiomyocyte cross sectional area after 14 and 30 days of HU, which partially reversed after 30 days of reloading [77]. Moreover, there was an increase in IMJ after 30 days, which reversed to control levels after the 30-day reloading period but increased even more after a second 14-day suspension period was added. This finding suggests a possible cumulative response to HU [78]. Meanwhile, right atrium cardiomyocytes isolated from male Wistar rats did not show any changes in cross sectional area after 14 and 30 days of HU, but increased after the 30-day reloading period, suggesting a late adaptation response [79]. Furthermore, there was an elevation in IMJ after 14 days but not after 30 days of HU [79].

#### 4.2.2. Sprague Dawley Rats

Sprague Dawley rats have also been exposed to HU. Female Sprague Dawley rats were exposed to HU for 7 days and showed a decrease in Ca^2+^ sensitivity of tension in ventricular cardiomyocytes [80]. Male Sprague Dawley rats were exposed to HU for 4 weeks, and left ventricular cardiomyocytes showed a decrease in myocardial function represented by decreases in left ventricular pressure, intracellular Ca^2+^ transient, L-type Ca^2+^ current, developed tension, shortening velocity, maximal isometric force, and Ca^2+^-activated ATPase activity [81,82]. A 4-week exposure also led to β-adrenoceptor desensitization and impaired adenylyl cyclase function, while β-adrenoceptor and G_Sα_-small protein levels remained unchanged, suggesting impaired post-receptor signaling in the G_S_-proteins/adenylyl-cyclase/cAMP cascade that contributes to the decreased cardiac contractility [83]. Another study using the same model and exposure duration investigated how HU affects cardiomyocyte apoptosis [84]. Apoptosis increased 1 day after reloading or via isoproterenol stimulation. In addition, an increase in calpain-2 activity and nuclear translocation was found after HU, and its inhibition prevented the apoptosis previously mentioned, suggesting that calpain-2 predisposes cardiomyocytes to apoptosis [84]. Finally, male Sprague Dawley rats examined under 4 weeks of HU also reported an increase in myocardial susceptibility to ischemia-reperfusion (IR) injury, as shown by increases in infarct sizes and enhanced apoptosis after IR [85]. It was also determined that AMP-activated protein kinase (AMPK) plays a key role, given that it decreased after IR, along with the p-AMPK/AMPK ratio, and treatment with an AMPK activator showed protective effects. Longer exposure (8 weeks) and administration of panax quinquefolium saponin (PQS), an AMPK activator, as a treatment found that PQS prevented the effects experienced during HU, including reduced heart weight, decreased left ventricular function, and ATP production, along with increased myocardial injury protein markers, fibrosis, and apoptotic index [51].

#### 4.2.3. Kunming Mice

Mice are also a widely used model in HU experiments. Male Kunming mice exposed to 28 days of HU experienced mitochondrial structural and functional changes, including increased mitochondrial number, more compact cristae, and increased ATP synthase and citrate synthase activity [86]. The results demonstrated an increase in apoptosis (elevated caspase-3 protein level and Bax/Bcl-2 ratios), mitochondrial autophagy (increased LC3II/LC3I ratio and phosphorylated parkin), and mitochondrial fusion (increased MFN1, MFN2, OPA1L proteins) [86].

#### 4.2.4. C57BL/6 Mice

One study exposed male C57BL/6 mice to 14–21 days of HU to determine its effects on the baroreflex-mediated control of HR, MAP, and cardiac contractility (inotropic response) [87]. HU caused a decrease in all these parameters in addition to a lower heart/body weight ratio, suggesting cardiac atrophy. Moreover, similar to Sprague Dawley rats, HU exposure led to β-adrenoceptor desensitization; however, Ca^2+^ transients were unaffected, suggesting impaired myofilament Ca^2+^ sensitivity [87]. Another study exposed male C57BL/6 mice to HU for either 28 or 56 days and reported ventricular dilation, reduced ejection fraction, and increased susceptibility to ventricular arrhythmias [88]. Additionally, abnormal Ca^2+^ handling was observed, including decreased sarcoplasmic reticulum Ca^2+^ content, increased spontaneous Ca^2+^ release events, and increased sarcoplasmic reticulum Ca^2+^ leak. CaMKII autophosphorylation was increased as well as CaMKII-dependent phosphorylation of RyR2, suggesting that Ca^2+^ handling and cardiac arrythmia development may be influenced by CaMKII signaling and RyR2 phosphorylation [88].

C57BL/6 mice with cardiomyocyte-specific knockout (KO) of calpain-1 were exposed to HU for 28 days and showed improved outcomes in heart size, cardiomyocyte size, fractional shortening, and ROS production compared to wild-type mice [89]. These data also showed that calpain promotes NADPH oxidase activation and ROS production via ERK1/2 and p38 phosphorylation. This suggests that calpain plays a major role in ROS production and cardiac dysfunction during HU, and its inhibition may counteract these effects [89]. Mice have also shown a reduced expression of CKIP-1 and miR-199a-3p after 4 and 6 weeks of HU, respectively [90,91]. The use of transgenic mice overexpressing CKIP-1 and miR-199a-3p, separately, prevented reductions in left ventricular mass, ejection fraction, and fractional shortening. Moreover, CKIP-1 overexpression protected against the increase in cardiac remodeling genes, such as *COL1A1*, *COL3A1*, and *BNP* [90]. miR-199a-3p also prevented the upregulation at the protein level of MEF2C, which is an important regulator of pathological cardiac remodeling following changes in pressure load [91]. Finally, WWP1-KO mice exposed to HU for 6 weeks preserved cardiac mass, cardiomyocyte size, ejection fraction, and fractional shortening parameters compared to the wild-type mice, which showed reductions in these parameters compared to controls [92]. WWP1-KO mice also prevented DVL2 protein accumulation and subsequent phosphorylation of CaMKII and HDAC4. These results suggest that WWP1 induces cardiac remodeling via the DVL2/CaMKII/HDAC4/MEF2C pathway [91,92].

#### 4.2.5. Pharmacological or Therapeutic Interventions

Multiple pharmacological or therapeutic interventions on HU-exposed have been reported. When exposed to 28 days of HU, C57BL/6 mice exhibited reductions in cardiomyocyte size, heart weight, ejection fraction, and fractional shortening [93]. Increases in angiotensin-II, oxidative stress, NADPH oxidase activation, and MuRF1, a muscle atrophy marker, expression were also reported. Moreover, the use of losartan, an angiotensin-II receptor blocker, significantly mitigated the effect of HU on these parameters, suggesting its possible use as a drug to prevent cardiac changes in astronauts on space [93]. Pharmacological inhibition of Rac1 using NSC23766 and atorvastatin during 28 days of HU on C57BL/6 mice also restored cardiomyocyte size, heart weight, fractional shortening, and creatine kinase levels [94]. Both inhibitors also prevented the increase in NOX2-containing NADPH oxidase activation, ROS production, and oxidative stress. Moreover, viral administration of miR-199a-3p prevented the effects of s-μg in mice after 42 days of HU [91]. Finally, time-restricted feeding (TRF) has also shown to be beneficial to alleviate cardiac dysfunction after 6 weeks of HU in male Sprague Dawley rats [95]. TRF reversed the reduced heart and body weight experienced during HU, along with the increased cardiomyocyte apoptosis and decreased left ventricular ejection fraction and fractional shortening. Additionally, TRF restored FGF21 signaling function, while blocking FGF21 signaling also blocked the protective effect of TRF, confirming the role of FGF21 as a mediator [95].

#### 4.2.6. Current Insights, Limitations, and Future Directions for the HU Model

Despite the differences in the species utilized, similar outcomes have been reported after HU exposure. Structural changes included altered mitochondrial structure and decreases in heart weight and cardiomyocyte cell size [51,77,87]. Consistent functional changes include reduced ejection fraction, fractional shortening, baroreflex dysfunction, and altered Ca^2+^ handling parameters [82,83,87,88]. Increases in apoptosis and oxidative stress have also been reported across the literature [84,85,86,89]. While many of these changes proved to be reversible upon return to normal conditions, they suggest that there are potential cardiac risks associated with μg.

In addition, therapeutic interventions and KO models helped elucidate the potential mechanisms through which HU acts. WWP1- and Calpain 1-KO mice, along with transgenic mice overexpressing CKIP-1 or miR-199a-3p, have shown to prevent the decreases in heart size, ejection fraction, and fractional shortening observed in wild type mice, indicating their essential role in cardiac remodeling under HU [89,90,91,92]. Moreover, the use of angiotensin-II receptor blockers as well as TRF or Rac1 inhibitors proved to be beneficial to counter the effects of HU [93,94,95]. These results highlight the opportunity to develop preventive strategies to protect cardiovascular health in space. Nonetheless, the HU model suffers from limitations that impair the extrapolation of results to human physiology under real μg conditions. While mimicking fluid shifts and the perceived volume overload experienced by astronauts in space, it is not clear if the fluid dynamics and mechanical forces deriving from the HU model are similar to those experienced under μg. Additionally, HU most likely induces stress responses on rodents which may be misinterpreted, adding noise and complexity to the results. Addressing these limitations in future experiments may lead to clearer and translatable outcomes that expand our knowledge of human physiology under real μg.

## 5. Conclusions

**Figure 3:**
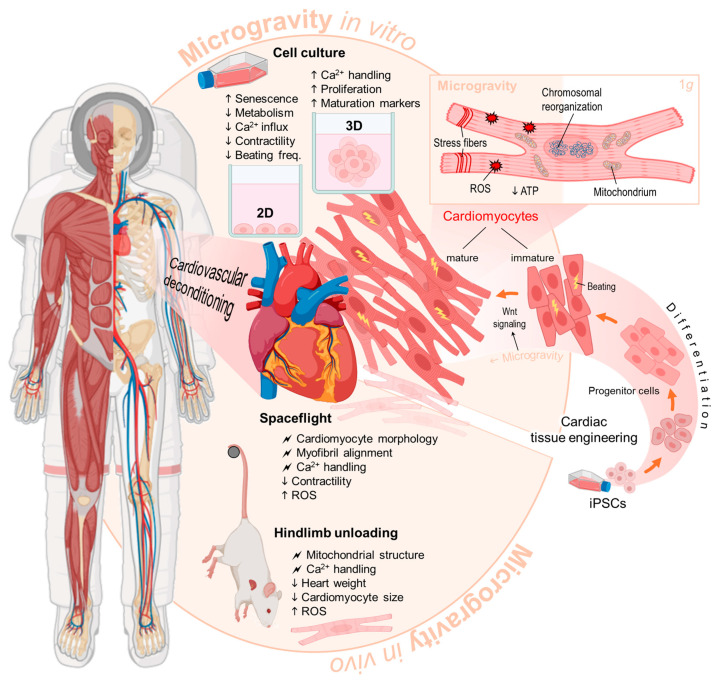
Effects of microgravity on cardiomyocytes in vitro and in vivo. iPSC: induced pluripotent stem cell, ROS: reactive oxygen species. ↑, increase; ↓, decrease; 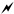, disturbing influence. Parts of the figure were drawn using pictures from Biorender.com and Servier Medical Art.

In conclusion, cardiac cell research under μg has made significant progress over the last 30 years (summarized in Figure 3). This review compiled and analyzed a large number of studies employing a large range of μg platforms, cardiac models, exposure durations, and outcome measurements. While the heterogeneity of the literature limits our ability to draw definitive conclusions, several trends and areas of opportunity emerge from this review. First, results show that μg exposure consistently produces changes in Ca^2+^ handling, contractility, metabolism, and gene expression. Whether these changes are beneficial or detrimental to cardiomyocytes depends on the choice of the cardiac model, μg platform, and exposure duration. When the aim is to understand the cellular and molecular changes underlying the systemic cardiovascular changes under μg or spaceflight conditions, researchers may benefit from identifying and standardizing cardiac models that better resemble the mechanical and chemical stimuli acting on cardiac cells during these conditions. This is the case also for animal models, which may benefit from a standardized and better characterized model with clear physiological relevance and limitations that allow researchers to draw meaningful and definitive conclusions. Second, 3D HiPSC-CM models under μg conditions have shown increased signs of tissue maturation and proliferation compared to their 1 g counterparts. These results open the venue for future research to combine the use of μg platforms with other tissue maturation strategies in order to unleash the therapeutic potential of HiPSC-CM against myocardial injury, among other cardiovascular diseases, which currently encounters many obstacles. Similarly, the stemness and increased cardiac development capacity CPCs and mESCs under μg conditions may help in the development of improved, more robust cardiac differentiation protocols.

## Figures and Tables

**Table 1:** Search strategy and number of articles found for each database.

Database	Search Strategy	No. of Articles
PUBMED	Cardiomyocytes AND (microgravity OR spaceflight)	150
Scopus	Cardiomyocytes AND (microgravity OR spaceflight)	47
Embase	Cardiomyocytes AND (microgravity OR spaceflight)	336
Google Scholar	allintitle: Cardiomyocytes microgravity OR spaceflight OR weightlessness	23
BIOSIS Previews	Cardiomyocytes AND (microgravity OR spaceflight)	20
	Total	576

**Table 2:** Inclusion and exclusion criteria for study selection.

Inclusion Criteria	Exclusion Criteria
Studies involving cardiomyocytes exposed to microgravity (real or simulated) or spaceflight conditions	Studies not involving cardiomyocytes
Articles published in peer-reviewed journals	Studies not involving microgravity or spaceflight conditions
Studies reporting outcomes regarding cardiac cell structure, function, OMICs, and other relevant endpoints	Review articles, editorials, and commentaries
Publications written in English	Articles not written in English

## Data Availability

The original contributions presented in this study are included in the article/Supplementary Materials. Further inquiries can be directed to the corresponding author.

## Supplementary Materials

The following supporting information can be downloaded at: https://www.mdpi.com/article/10.3390/biom15091261/s1, Table S1: Studies under real microgravity conditions; Table S2: In vitro studies under simulated microgravity conditions; Table S3: Studies under hindlimb unloading model.

## Abbreviations

The following abbreviations are used in this manuscript:

μg	Microgravity
SV	Stroke volume
CO	Cardiac output
CVP	Central venous pressure
DBP	Diastolic blood pressure
MAP	Mean arterial pressure
SBP	Systolic blood pressure
HR	Heart rate
ANP	Atrial natriuretic peptide
SVR	Systemic vascular resistance
HRV	Heart rate variability
POI	Postflight orthostatic intolerance
HiPSC-CMs	Human-induced pluripotent stem cell-derived cardiomyocytes
ISS	International space station
CSS	Chinese space station
TCA	Tricarboxylic acid
s-μg	Simulated microgravity
ECM	Extracellular matrix
dECM	Decellularized porcine myocardial extracellular matrix
rGO	Reduced graphene oxide
CPCs	Cardiovascular progenitor cells
mESCs	Mouse embryonic stem cells
EBs	Embryoid bodies
AC5	Adenylyl cyclase type 5
RWV	Rotating wall vessel
ROS	Reactive oxygen species
CaMKII	Ca^2+^/calmodulin-dependent protein kinase II
HDAC4	Histone deacetylase 4
BNP	Brain natriuretic peptide
SSR	Simulated space radiation
IMJ	Intramitochondrial junction
IR	Ischemia–reperfusion
AMPK	AMP-activated protein kinase
PQS	Panax quinquefolium saponin
TRF	Time-restricted feeding
