# Supplementary material for: The Effects of Microgravity on the Structure and Function of Cardiomyocytes
Source: Biomolecules. 2025 Aug 30;15(9):1261. doi: 10.3390/biom15091261 (PMC12467004; doi:10.3390/biom15091261)
Supplement: Supplementary file 1 [file biomolecules-15-01261-s001.zip › biomolecules-3812798-supplementary.pdf]

biomolecules

MDPI

Table S1. Studies under real microgravity conditions.

Year	Cell line/ Animal species	Microgravity platform	Control	Duration	Intervention	Outcomes	DOI
2019	HiPSC-CMs	Real μg aboard a parabolic flight	Ground control on Earth	31 alternating phases of hypergravity and μg lasting around 3.5 hours in total	Pre-treatment with isoprenaline (ISO), a β-adrenoceptor agonist, bay-K8644 (Bay-K), an L-type calcium channel (LTCC) agonist and nifedipine, an LTCC blocker	Beating rate (BR) increased after 15th parabola and persisted until one hour after the flight. Pre-treatment with nifedipine completely prevented this increase.Both ISO and Bay-K significantly increased BR during ground control conditions, and these effects were further amplified by an additional 20–25% after exposure to 25 or more parabolas.	10.3390/cells8040352
2012	Male C57BL/6 mice	Real μg aboard a parabolic flight	Mice treated with phosphate buffered saline	8 – 12 alternating phases of hypergravity and μg lasting 15-20 seconds each	Treatment with NKH477, a selective activator of adenylyl cyclase type 5 (AC5), and vidarabine, an inhibitor of AC5	Mice treated with NKH477 showed more stable HR and HR variability during flight in comparison to control and to mice treated with vidarabine. Treatment with NKH477 increased ratio of high frequency power.	10.1254/jphs.12102FP
2008	Transgenic mice with either overexpressed (AC5TG) or disrupted (AC5KO) AC5	Real μg aboard a parabolic flight	Wild-type (WT) control	Alternating phases of hypergravity and μg lasting 15-20 seconds each	N/A	AC5KO mice expression showed higher heart rate variability (HRV) during microgravity, with a significant increase in the standard deviation of normal R-R intervals. AC5TG mice exhibited reduced HRV. AC5KO mice led to impaired autonomic regulation of heart rate, while AC5 overexpression provided stabilizing effects.	10.1152/japplphysiol.01166.2007
1999	Sprague-Dawley pregnant rat, Sprague-Dawley fetus	Real μg aboard aboard NASA space shuttles	Ground control on Earth	11 days	N/A	Increase in ANP mRNA expression, but decrease in ANP protein content. No conclusive data on adult pregnant rats, but tendency shows an increase in cardiac ANP biosynthesis.	10.1016/s0024-3205(99)00090-9
1992	Wistar male rats	Real μg aboard COSMOS 2044 Biosputnik	Ground control on Earth	2 weeks	N/A	Reduced cross sectional area of myofibrils for papillary muscles. Overall morphology of the tissue suggests adequate preservation and no major damage to cardiac tissue.	10.1152/jappl.1992.73.2.S94
2014	Male C57BL/6 mice	Real μg aboard the BION-M1 biosatellite	Vivarium and synchronous control on Earth	30 days	N/A	Reduction in cytoplasmic fraction β-actin and membranous fraction α-actinin 4 in CMs from the left ventricle. Downregulated mRNA levels of β-actin (*ACTB*) and α-actinin 4 (*ACTN4*) and upregulated α-actinin 1 (*ACTN1*).	10.1152/japplphysiol.00134.2014
2024	HiPSC-CMs	Real μg aboard the CSS	Simulated 1*g* conditions on the CSS	6 days	Thiamine supplementation	Impaired thiamine utilization, affecting TCA cycle and reducing ATP production. 13 dysregulated metabolites mainly involved in the sulfur relay system, thiamine metabolism, ABC transporters, and vitamin digestion and absorption. Decreases in sarcomere length, cardiomyocyte size, cTnT content, contractile function and slower Ca^{2+} cycling. Changes in cardiomyocyte-specific genes (*TNNI1*, *TNNI3*, *ATP2A2*, and *CACNA1C*). Thiamine supplementation	10.1038/s41392-024-01791-7

						under s-μg improved ATP production, Ca^{2+} handling, and restored TCA cycle function and sarcomere structure.	
2020	Drosophila melanogaster (fruit flies)	Real μg aboard the ISS	Ground control on Earth	30 days	N/A	Cardiac constriction, reduced heart chamber size, diminished cardiac output, and myofibrillar and ECM remodeling. Reduced mRNA expression of actin, myosin, and ECM components (*COLIV*, *MMP1*, *MMP2*).	10.1016/j.celrep.2020.108445
2021	Human Islet-1+ cardiovascular progenitor cells (CPCs) from adult and neonatal sources	Real μg aboard the ISS	Ground control on Earth	30 days	N/A	Increase in cardiac commitment markers, MESP1 and NKX2-5, regardless of age, as well as in stemness related markers. Also, pathway analysis revealed that Wnt/β-catenin, Ca^{2+} Signaling, Notch, ERBB, and Hippo pathways were induced regardless of age. Regardless of age, genes related to specific biological processes such as cell cycle progression, proliferation, differentiation, heart development, oxidative stress protection, and focal adhesion were induced.	10.3390/ijms22073577
2018	Human neonatal CPCs isolated from neonatal heart tissue	Real μg aboard the ISS	Ground control on Earth	30 days	N/A	Ca^{2+} signaling-related genes, *PLCG1, PRKCA,* and *CAMK2A* were upregulated. Gene and protein expression changes confirmed the activation of protein kinase C α (PKCα) and Akt.	10.1089/scd.2017.0263
2019	HiPSC-CMs	Real μg aboard the ISS	Ground control on Earth	5.5 weeks	N/A	Alterations in Ca^{2+} handling parameters, including increases in the transient decay tau and the standard deviation of beating intervals, suggesting decreased Ca^{2+} recycling rate and beating irregularity, respectively. No effect on sarcomere structure, length, or regularity. Changes in genes related to hypertrophy (*MEF2D, HDAC10, HDAC4, HDAC8*) and Ca^{2+} handling and contraction (*TNNT2*, *TNNI1*).	10.1016/j.stemcr.2019.10.006
2022	HiPSC-CMs	Real μg aboard the ISS, 3D cardiac progenitor spheres	Simulated 1g conditions on the ISS	3 days and 3 weeks	N/A	Increased cell size, spheroid size, Ca^{2+} handling kinetics, percentage of Ki67-positive cells, and proliferation. Increased expression of proliferation markers *(CCND1, CCND2, IGF2, TBX3).* Increased expression of cardiac structural genes (*MYL2, MYL7, TNNI3, TNNT2, MYH6, MYH7*). Improved Ca^{2+} handling: Increased peak amplitude, maximum rise slope and maximum decay slope, decreased time to peak and half-width, and upregulation of *CASQ2* and *ATP2B4*.	10.1016/j.stemcr.2022.08.007
2023	HiPSC-CMs	Real μg aboard the ISS, 3D cardiac progenitor spheres	Simulated 1g conditions on the ISS	3 weeks	N/A	Increased expression of proliferation and differentiation genes (*CCBE1, CCND2, IGFBP5, MYL2, TNNI3, ATP2B4, RYR2*). Downregulation of ECM genes (*COL4A4, ITGA11, ANXA1*). Enhanced cardiac function (Upregulation in *THBD, GADL1, ATP2B4, RYR2, PRKACA*).	10.1038/s41526-023-00336-6

2025	HiPSC-CMs	Real μ*g* aboard the ISS, 3D cardiac spheroids	Ground control on Earth	8 days	N/A	Contractility and Ca^{2+} transients remained unchanged. Upregulated proteins related to cell survival, proliferation and metabolic pathways ((UGT2A3, EEF2K, KRT13, PRIM1). Upregulated genes related to metabolism, mitochondria and cardiac development and structure (*TNNT2, PLCB2, CPNE5, CDH17, SFRP5, PLA2G4F, GPAT2*).	10.1016/j.biomaterials.2024.123080
2025	HiPSC-CMs	Real μ*g* aboard the ISS, heart-on-a-chip platform	Ground control on Earth	30 days	N/A	Structural damage, including shortened sarcomeres and fragmented mitochondria. Reduced contractile twitch force and increased arrhythmic activity. Downregulation of contractility-related genes (*MYL7, MYL3, MYH7B, TNNI3, RYR2, ATP2A2, TTN*) and upregulation of inflammation and oxidative stress-related genes (*NOXO1, GGTLC3, IL18, TGFB2*).	10.1073/pnas.2404644121
2015	Male C57BL/6N mice	Real μ*g* aboard the Russian spacecraft “BION-M1”	Ground control on Earth	30 days	N/A	Increase in titin mRNA expression and titin phosphorylation levels. Sarcomeric structure disruptions.	10.1155/2015/104735
2008	Neonatal rat cardiomyocytes	Real μ*g* aboard the Shenzhou-6 spacecraft	Ground control on Earth	115 hours	N/A	Cardiomyocytes showed disordered contraction with reduced synchrony. Reduction in ANP secretion. Disassembly of microtubules. No changes in F-actin.	10.1007/s11434-008-0167-y
2015	Mouse embryonic stem cells	Real μ*g* aboard the Space Shuttle Discovery	Ground control on Earth	15 days	N/A	Higher expression of stemness-related markers and reduced capacity to differentiate into different tissue types and to express terminal differentiation markers. Exhibited alterations in cell cycle, p53, Notch and Wnt signaling pathways. Showed twice the potential to differentiate into beating cardiomyocyte clusters upon returning to Earth.	10.1089/scd.2015.0218
2019	Mouse induced pluripotent stem cells	Real μg aboard the Tianzhou-1 cargo spacecraft	Ground control on Earth	2 weeks	N/A	Accelerated and more robust cardiomyocyte differentiation.	10.1089/scd.2018.0240

Table S2. In vitro studies under simulated microgravity conditions.

Year	Cell Line/ Animal species	Microgravity	Control	Duration	Intervention	Outcomes	DOI
2019	Cardiomyocytes isolated from male C57BL/6 mice	Simulated space radiation with either 900 mGy of protons or 150 mGy of 56Fe ions.	Cardiomyocytes from non-irradiated mice	Five time points up to 28 days after irradiation.	N/A	FYN gene found to be the overall central driver for the cardiovascular response when considering all the different experimental groups together. ROS pathways were downregulated in response to both protons and real spaceflight conditions. Molecular changes were observed even after 28 days, suggesting persistent effects of space radiation.	10.3390/ijms20030661
2018	Primary cardiomyocytes derived from chicken embryos.	Simulated space radiation with heavy ions (iron, titanium, and carbon) in an ion entrance channel setup	Sham-irradiated cardiomyocytes.	3 hours after irradiation and followed up for 24 hours	N/A	Efficient DNA damage repair after irradiation, even at high doses, with almost complete recovery of DNA double-strand breaks within 24 hours. Dose-dependent reduction in cell proliferation, but apoptosis levels remained low across all doses. Slight dose-dependent decrease in beating rate after titanium ion exposure and prolonged field action potential duration after higher doses.	10.1016/j.lssr.2018.01.001

2023	Engineered human cardiac tissues	Simulated space radiation with neutrons (1 Gy) or photons (4 Gy) using a terrestrial galactic cosmic ray simulator	Non-irradiated engineered cardiac tissues.	Up to 3 weeks after irradiation	Treatment with radioprotective agent amifostine	Neutron-irradiated tissues showed increased force generation and contractility, indicating early hypertrophic response. Increased expression of stress-response genes (e.g., HMOX1) and genes involved in cardiac hypertrophy.	10.1016/j.biomaterials.2023.122267
2023	Human-induced pluripotent stem cell-derived cardiomyocytes (HiPSC-CMs)	s-μ*g* using a rotating wall vessel (RWV), cells were attached on aligned nanofibers	1*g* control	1,3, 5 days	N/A	The projected area of tissue decreased from d0 to d3, creating thicker tissue. Changes in nuclei shape and F-actin distribution. Increased formation of cardiac-specific proteins Lamin A/C, β-MHC and Connexin-43 significantly after d3, indicating tissue maturation.	10.1016/j.reth.2023.09.002
2022	HiPSC-CMs	s-μ*g* using a 2D Clinostat	1*g* control	48 hours	N/A	Increased ROS production, mitochondrial function impairment, slower Ca^{2+} influx, reduced contraction velocity, upregulation of senescence markers, and altered chromatin organization and 3D genome organization.	10.1016/j.isci.2022.104577
2020	HL-1 cardiomyocytes	s-μ*g* using a 2D Clinostat	1*g* control	48 hours	SiRNA-CaMKII	Increased basal cytosolic Ca^{2+}, Ca^{2+} oscillations, Ca^{2+} transients, phosphorylation of CaMKIIδ (Thr287) and HDAC4 (Ser632), expression of ANP and BNP. Decreased α-MHC and cell size. SiRNA-CaMKII prevented cell size change and phosphorylation of HDAC4, indicating that the CaMKII/HDAC4 pathway may be involved in the cardiac remodelling during s-μ*g*.	10.1111/cpr.12783
2007	HL1 cardiomyocytes, cardiac fibroblasts, and resident cardiac stem cells.	s-μ*g* using a 2D Clinostat	1*g* control	1, 24, 72 hours	N/A	Increased apoptosis and modifications in cytoskeleton, cell-adhesion components and proliferative ability after 1- , 24- and 72-hours. When reconditioning conditions were applied, only resident cardiac stem cells recovered cell proliferation ability.	10.1016/j.yjmcc.2007.03.198
2002	Primary cardiac myocytes from 2-day old Wistar rats	s-μ*g* using a 2D Clinostat	1*g* control	8, 24, 36, and 48 hours	Staurosporine (non-selective protein kinase C (PKC) inhibitor) and calphostin C (selective PKC inhibitor)	Increased NO levels, iNOS mRNA and iNOS protein expression. Inhibitors decreased NO levels, but not back to the 1*g* control level. Calphostin C indicated that PKC contributed to iNOS regulation.	10.1360/03yc9032
2016	Mouse embryonic stem cells	s-μ*g* using a 2D pipette clinostat; embryoid bodies (EBs) in suspension	1*g* control	3 days of s-μ*g* followed by 7 days of 1*g* for further differentiation	N/A	Downregulation of cardiac-specific genes (*TNNT2, RBP4, TNNI1, CSRP3, NPPB, MYBPC3*) after 10 days, indicating cardiac developement inhibition. Decrease in EBs beating rate.	10.1159/000443090
2009	H9C2 cardiomyocytes	s-μ*g* using a rotating wall vessel (RWV); cells attached on the surface of microcarrier beads	Stationary 1*g* control and dynamic 1*g* control	3 hours	N/A	50% increase in NF-kB subunit p65 nuclear translocation	10.1115/1.3128718

1997	Neonatal rat heart cells	s-μ*g* using a RWV; cells cultured in microcarrier beads	1*g* control	6 days	N/A	3D multilayered growth patterns and increased oxidative metabolism, indicated by elevated levels of NAD-dependent cytochrome-c reductase and slightly reduced levels of creatine kinase.	10.1007/s11626-997-0003-8
2016	Primary rat neonatal cardiomyocytes	s-μ*g* using a RWV; cells in suspension	1*g* control	12, 48, 120 hours	N/A	Reduced protein synthesis evidenced by decreased protein turnover. Upregulation of proteins involved in mitochondrial maintenance and protection (e.g., mortalin and AFG3L2). Activated endoplasmic reticulum stress and unfolded protein response, but preserved mitochondrial function.	10.1038/srep34091
2021	H9C2 cardiomyocytes	s-μ*g* using an RPM	1*g* control	6, 12, 24, 48, 72, 96 hours	1mM of N-acetyl-cysteine (antioxidant)	Morphological changes with increases in cell height, actin filament mean length, and altered mitochondrial branching complexity. Increased levels of cytosolic Ca^{2+}, medium glucose, medium lactate, ROS and mitochondrial O_2^- (1mM of N-acetyl-cysteine prevented this). Decreased mitochondrial potential and metabolic activity	10.1155/2021/9951113
2024	HiPSC-CMs	s-μ*g* using an RPM	1*g* control	7, 14 days	N/A	Enhanced the maturation of HiPSC-CMs after 7 day with increased sarcomere length, increased z-disc length. Improved mitochondrial characteristics with increased mitochondrial content, mitochondrial membrane potential, and ATP production. Enhanced Ca^{2+} transients and kinetics.	10.1038/s41598-024-52453-1
2016	IMR90 iPSCs, H7 and H9 human embryonic stem cells	s-μ*g* using an RPM	1*g* control	3 days during day 5 of differentiation process	N/A	Increased cardiomyocyte yield by 1.5- to 4-fold compared to controls and improved Ca^{2+} handling, with faster Ca^{2+} reuptake. Upregulated genes related to cardiomyocyte structural proteins (*MYH6, MYH7, MYL2, MYL7, TNNI1, TNNI3*), calcium-handling proteins (*ATP2A2, CASQ2, RYR2, SLC8A1*), survival (*BIRC5, HSP60, HSP70, HSP90*), proliferation (*MKI67, PCNA*), and cell cycle (*AURKA, AURKB, CCNB1*).	10.1038/srep30956
2018	iPSCs derived from mouse models (including cKitCreERT2/+, Isl1nLacZ, Wnt1-Cre reporter alleles)	s-μ*g* using the rotary cell suspension culture system (RCCS)	1*g* control	10 to 21 days	N/A	s-μ*g* exposure favors the growth of mesodermal cardiac progenitors over neural crest derived autonomic neurons and cardiomyocytes. Reduction in the rate of spontaneously beating EBs on day 10 of the differentiation process.	10.1089/scd.2017.0265

Table S3. Studies under hindlimb unloading model.

Year	Rodent model	Control	Duration	Intervention	Outcomes	DOI
1996	Female Sprague-Dawley rats	1*g*	1 week	N/A	Decreased Ca2+ sensitivity of tension in ventricular cardiomyocytes	10.1152/jappl.1996.80.5.1612
2005	Male C57BL/6 mice	1*g*	2, 3 weeks	N/A	Reduced heart rate, mean arterial pressure, cardiac contractility (inotropic response), heart/body weight ratio. β-adrenoceptor desensitization.	10.1152/ajpheart.01091.2004

2019	Male C57BL/6 mice	1g	4 weeks	Treatment with losartan, an AT1R blocker, and apocynin, a selective NADPH oxidase inhibitor	Reduced cardiomyocyte size, heart weight, ejection fraction and fractional shortening. Increased angiotensin-II, oxidative stress, NADPH oxidase activation and MuRF1 expression. Losartan significantly mitigated the effect of HU on these parameters.	10.1186/s12967-019-2021-1
2022	Male C57BL/6 mice	1g	4 weeks	Treatment with NSC23766 and atorvastatin, Rac1 inhibitors	Treatment with NSC23766 and atorvastatin restored cardiomyocyte size, heart weight, fractional shortening, and creatine kinase levels, which were altered after HU.	10.1007/s12265-021-10197-7
2014	Male C57BL/6 mice	1g	4, 8 weeks	N/A	Ventricular dilation, reduced ejection fraction, and increased susceptibility to ventricular arrhythmias. Abnormal Ca^{2+} handling including decreased sarcoplasmic reticulum Ca^{2+} content, increased spontaneous Ca^{2+} release events and sarcoplasmic reticulum Ca^{2+} leak. Increased CaMKII autophosphorylation and CaMKII-dependent phosphorylation of RyR2.	10.1016/j.ijcard.2014.08.138
2020	Male C57BL/6 mice with knockout (KO) of Calpain-1	1g	2, 4 weeks	N/A	Improved heart size, cardiomyocyte size, fractional shortening, and ROS production compared to wild-type mice under HU. Calpain was shown to promote NADPH oxidase activation and ROS production via ERK1/2 and p38 phosphorylation.	10.1074/jbc.RA119.011890
2021	Male C57BL/6 mice with KO of WWP1	1g	6 weeks	N/A	WWP1-KO mice preserved cardiac mass, cardiomyocyte size, ejection fraction and fractional shortening parameters compared to the wild-type mice after HU. WWP1-KO mice prevented DVL2 protein accumulation and subsequent phosphorylation of CaMKII and HDAC4.	10.3389/fcell.2021.739944
2023	Male Kunming mice	1g	2, 4 weeks	N/A	Increased mitochondrial number, more compact cristae, and increased ATP synthase and citrate synthase activity. Increase in apoptosis (elevated caspase-3 protein level and bax/bcl-2 ratios), mitochondrial autophagy (increased LC3II/LC3I ratio and phosphorylated parkin) and mitochondrial fusion (increased MFN1, MFN2, OPA1L proteins).	10.1113/EP090518
2001	Male Sprague-Dawley Harlan rats	1g	1, 2, 3, 4 weeks	N/A	Decreased myocardial function,developed tension, velocity of shortening, Ca^{2+} activated ATPase activity of cardiac myofibrils, and maximal isometric force of skinned cardiac muscle. Upregulated NH2-terminal truncated cTnI fragment.	10.1074/jbc.M0110448200
2008	Male Sprague-Dawley rats	1g	4 weeks	N/A	β-adrenoceptor desensitization and impaired adenylyl cyclase function. β-adrenoceptor and Gsα-small protein levels remained unchanged, suggesting impaired post-receptor signaling in the GS-proteins/adenylyl-cyclase/cAMP cascade that contributes to decreased cardiac contractility.	10.1152/japplphysiol.01381.2007
2010	Male Sprague-Dawley rats	1g	4 weeks	N/A	Reduced cardiac contractility, responsiveness to β-adrenoceptor stimulation, left ventricular pressure, systolic function, intracellular Ca^{2+} transient and L-type Ca^{2+} current. Impaired post-receptor signaling in β-adrenoceptor/Gs protein/adenylyl cyclase/cAMP/PKA/Ca^{2+} cascade that contributes to decreased cardiac contractility.	10.1152/japplphysiol.01055.2009
2011	Male Sprague-Dawley rats	1g	4 weeks	Treatment with isoproterenol, a β-adrenergic agonist, propranolol, a β-blocker, and PD150606, a calpain inhibitor.	Apoptosis increased 1 day after reloading or via isoproterenol stimulation. In addition, increases in calpain-2 activity and nuclear translocation were found after HU, and its inhibition prevented the apoptosis previously mentioned, suggesting that calpain-2 predisposes cardiomyocytes to apoptosis.	10.1002/jcb.22947

2017	Male Sprague-Dawley rats	1*g*	4 weeks	Ischemia-Reperfusion after HU and treatment with A-769662, an AMPK activator	Increases in infarct sizes and enhanced apoptosis after ischemia-reperfusion (IR), suggesting increased myocardial susceptibility to IR injury after HU. Decreased AMPK and p-AMPK/AMPK ratio after IR, and treatment with A-769662 showed protective effects.	10.1139/cjpp-2015-0456
2020	Male Sprague-Dawley rats	1*g*	6 weeks	Time-restricted feeding (TRF)	TRF prevented the increased cardiomyocyte apoptosis and the reduced left ventricular ejection fraction, fractional shortening and heart and body weight experienced after HU. TRF prevented FGF21 signaling dysfunction and blocking FGF21 signaling also blocked the protective effect of TRF, confirming the role of FGF21 as a mediator.	10.1096/fj.202001246RR
2019	Male Sprague-Dawley rats	1*g*	8 weeks	Treatment with panax quinquefolium saponin (PQS), an MAPK activator	PQS prevented the reduced heart weight, decreased left ventricular function and ATP production experienced after HU. Also prevented the increased myocardial injury protein markers, fibrosis and apoptotic index observed during HU.	10.1016/j.phymed.2018.08.007
2025	Male transgenic mice overexpressing miR-199a-3p	1*g*	6 weeks	N/A	Transgenic mice overexpressing miR-199a-3p prevented the reductions in left ventricular size, ejection fraction, and fractional shortening. Also prevented upregulation of MEF2C protein levels.	10.1096/fj.202402248R
2024	Male Wistar rats	1*g*	1 week	N/A	Gene expression changes in phosphodiesterase (*PDE2A, PDE3A, PDE4A, PDE4B*), soluble guanylate cyclase (*GUCY1A1, GUCY1A2, GUCY1B1*) and adenylate cyclase (*ADCY5, ADCY6*) isoforms, which are volved in pathways that regulate mechanosensitive and mechanically gated channels.	10.1007/s10517-024-06024-z
2023	Male Wistar rats	1*g*	1 week	N/A	Gene expression changes in mechanically gated (*TRPM7, TRPP1, TRPP2, PIEZO1, TMEM63A, TMEM63B, TRPV2*) and mechanosensitive (*KCNK2, KCNK3, KCNJ11, KCNJ8, SCN5A, CACNA1C, KCNQ1*) ion channels	10.1134/S1607672923700369
2012	Male Wistar rats	1*g*	1, 3, 7, 14 days HU, and 3, 7 days 1*g* after 14 days HU.	N/A	Increase in transversal stiffness of the contractile apparatus in multiple locations from 3 to 14 days of HU, but reversed after 7 days of 1*g*. Increased respiration rate parameters, desmin protein content and membranous γ-actin protein content from 1 to 14 days HU, but also reversed after 7 days of 1*g*. Elevated cytoplasmic α-actinin-4 protein content from 1 to 14 days of HU.	10.1155/2012/659869
2003	Male Wistar rats	1*g*	14, 30 days, 30 days 1*g* after 30 days HU, 14 days HU after 30 days 1*g* after 30 days HU	N/A	No changes in cross sectional area after 14 and 30 days of HU, but increased after the 30 days of 1*g*. Increased IMJ after 14 days but not after 30 days of HU	10.1023/B:BIBU.0000014350.53367.b7
2003	Male Wistar rats	1*g*	14, 30 days, 30 days 1*g* after 30 days HU, 14 days HU after 30 days 1*g* after 30 days HU	N/A	Decreased left ventricle cardiomyocyte cross sectional area after 14 and 30 days, which partially reversed after 30 days of reloading.	10.1023/a:1026108812322
2004	Male Wistar rats	1*g*	14, 30 days, 30 days 1*g* after 30 days HU, 14 days HU after 30 days 1*g* after 30 days HU	N/A	Increased IMJ after 30 days, reversed to control levels after a 30-day reloading period, but increased even more after a second 14-day suspension period was added, suggesting a possible cumulative response.	10.1023/b:bebm.0000031573.88461.94
2010	Male Wistar rats	1*g*	24 days	N/A	5% increase in the left ventricle cardiomyocyte cross sectional area, and an 11.5% increase in relative number of capillaries per cardiomyocyte	10.1134/S001249

						66100 40149
1994	Male Wistar rats	1*g*	1/4, 1/2, 3/4, 1, 2, 6 hours	N/A	Increase in ANP plasma levels after 1 hour that reversed to baseline after another hour, and remained constant for the next 4 hours.	10.10 16/01 67- 0115(94)90 539-8
2012	Male Wistar rats	1*g*	24 days	N/A	Increased intramitochondrial junctions	10.11 34/S1 06235 90120 30132
2018	Transgenic mice overexpressing CKIP-1	1*g*	4 weeks	N/A	Transgenic mice prevented reductions in left ventricular mass, ejection fraction, and fractional shortening observed in wild-type mice after HU. CKIP-1 over-expression protected against increases in cardiac remodeling genes (*COL1A1, COL3A1, BNP*).	10.33 89/fp hys.2 018.0 0040
